# Construct validity of real-world digital mobility outcomes in patients after proximal femoral fracture: a cross-sectional observational study

**DOI:** 10.1038/s41598-026-43297-y

**Published:** 2026-03-20

**Authors:** Tobias Eckert, Martin Aursand Berge, Michael Long, Martí de las Heras, Paula Alvarez, Hubert Blain, Julia Braun, Joren Buekers, Brian Caulfield, Monika Engdal, Anja Frei, Jorunn L. Helbostad, Anisoara Ionescu, Carl-Philipp Jansen, Lars Gunnar Johnsen, Jochen Klenk, Sarah Koch, Vita Lanfranchi, Lynn Rochester, Clemens Becker, Beatrix Vereijken, Judith Garcia-Aymerich

**Affiliations:** 1 Robert Bosch Foundation for Medical Research, Stuttgart, Germany; 2 Geriatric Center, Medical Faculty Heidelberg, Heidelberg University, Heidelberg, Germany; 3 Department of Neuromedicine and Movement Science, Norwegian University of Science and Technology, Trondheim, Norway; 4 Department of Computer Science, University of Sheffield, Sheffield, UK; 5 ISGlobal, Barcelona, Spain; 6 Universitat Pompeu Fabra (UPF), Barcelona, Spain; 7 CIBER Epidemiología y Salud Pública (CIBERESP), Barcelona, Spain; 8 University Hospital of Montpellier, Montpellier, France; 9 Epidemiology, Biostatistics and Prevention Institute, University of Zurich, Zurich, Switzerland; 10 School of Public Health, Physiotherapy & Population Science, University College Dublin, Dublin, Ireland; 11 Insight Research Ireland Centre For Data Analytics, University College Dublin, Dublin, Ireland; 12 École Polytechnique Fédérale de Lausanne, Lausanne, Switzerland; 13 Department of Orthopaedic Surgery, St. Olav’s Hospital, Trondheim, Norway; 14 Institute of Epidemiology and Medical Biometry, Ulm University, Ulm, Germany; 15 Study Center Stuttgart, IB University of Health and Social Sciences, Stuttgart, Germany; 16 Department of Sport, Exercise, and Health, University of Basel, Basel, Switzerland; 17 Translational and Clinical Research Institute, Newcastle University, Newcastle Upon Tyne, UK; 18 Newcastle Biomedical Research Centre (BRC), National Institute for Health and Care Research (NIHR), Newcastle University and The Newcastle upon Tyne Hospitals NHS Foundation Trust, Newcastle Upon Tyne, UK

**Keywords:** Digital mobility outcomes, Wearable device, Walking, Validity, Hip fracture, Diseases, Health care, Medical research

## Abstract

Digital mobility outcomes (DMOs) offer unique insights into recovery of real-world mobility after proximal femoral fracture (PFF), but their clinical validity remains to be established. This study assessed construct validity (convergent, divergent, and known-groups) of 24 DMOs measuring walking activity (amount, pattern) and gait (pace, rhythm, bout-to-bout variability) in patients within one year after PFF. Patients were recruited from inpatient and outpatient lists at five European sites, resulting in 505 included participants (66% female), with mean age of 77.6 ± 9.4 years and supervised gait speed of 0.7 ± 0.4 m/s. Mobility was monitored over seven days using a single wearable device on the lower back. Convergent and divergent validity analyses were stratified by two groups: acute (≤ 14 days since surgery) and non-acute (≥ 15 days since surgery). Correlations between DMOs and related (clinical- and patient-reported mobility outcomes) and unrelated constructs (hearing impairment and systolic blood pressure) were compared to a priori expected correlations. Known-groups validity was assessed across four recovery phases. The results were evaluated individually by experts and in a subsequent consensus meeting, with 17 of 24 DMOs showing evidence of construct validity in non-acute PFF patients. These findings represent an initial step in a larger process towards regulatory endorsement.

**Supplementary Information:**

The online version contains supplementary material available at 10.1038/s41598-026-43297-y.

## Introduction

Proximal femoral fractures (PFF) in older adults represent a life-changing clinical event with high one-year mortality rates ranging between 20 and 30%^1^. Many patients experience long-term functional decline, with high rates of care home admission and only 40–60% regaining their pre-fracture mobility in extended activities of daily living^2^. Independent mobility plays a central role in social participation and quality of life after a PFF^3^, making recovery of walking ability a key rehabilitation target. Mobility recovery evolves over the first postoperative year, beginning with an *acute* phase focused on surgery, mobilisation and pain management^4^. This is followed by a *post-acute* rehabilitation phase, aimed at restoring physical function and preparing patients for discharge^5^. Recovery then continues in an *extended* phase during the first six months, and ultimately a *long-term* phase in the latter half of the year, when mobility tends to stabilise^6–9^. Tracking mobility recovery across these phases may help clinicians identify mobility limitations and potential risks, and guide patient treatment and rehabilitation.

Traditionally, mobility has been measured with clinical tests under standardised, supervised conditions, offering recovery snapshots that may not capture how people move in daily life with changing environments^10^. Further, clinical tests are susceptible to floor and ceiling effects as well as reactivity to observation^11,12^. Wearable devices equipped with inertial measurement units offer a more differentiated and ecologically valid insight into patients’ mobility in real-world environments^13,14^. These devices enable the quantification of various walking-related digital mobility outcomes (DMOs)^14^ in walking domains such as amount, pattern, pace, rhythm and variability of walking^15^. However, research on real-world DMOs in older adults after PFF has been limited largely to the amount domain, e.g. number of steps per day and daily active time^16,17^. Due to technical complexity, spatial and temporal parameters that provide information on other domains of mobility, such as activity patterns (e.g., number of walking bouts), pace (e.g., walking speed) or rhythm (e.g., cadence) have rarely been measured in the real world after a PFF^18^. Recent methodological advances now make it possible to address this gap.

Building on a core set of technically validated measures, the Mobilise-D project created a processing pipeline that enables the estimation of 24 DMOs that reflect walking activity (amount- and pattern domains) and gait (pace, rhythm and bout-to-bout variability domains) across health conditions such as Parkinson’s disease (PD), chronic obstructive pulmonary disease (COPD), multiple sclerosis (MS) and older adults after a PFF^19–21^. Although valid in terms of criterion validity, the accuracy and reliability of some DMOs were lower in patients with more pronounced mobility limitations, such as those with slow walking speeds and gait irregularities^19–21^. These challenges in assessing real-world gait in populations with impaired gait function, together with the current lack of studies validating DMOs specifically in patients after a PFF^15^, emphasise the need for clinical validation. This is currently being addressed by the Mobilise-D consortium through a comprehensive assessment of the DMOs’ construct validity, ability to detect change, and predictive capacity, with the first descriptive results from the PFF cohort recently released^22^. This broad assessment lays the groundwork towards endorsement of DMOs by regulatory authorities.

This study assesses the construct validity of 24 real-world walking activity and gait DMOs in a large, heterogeneous, international sample of patients recruited within the first year after PFF surgery. We apply a comprehensive framework of construct validity following the COSMIN guidelines^23^, encompassing convergent validity (moderate-to-strong associations with established mobility measures), divergent validity (weak or absent associations with unrelated constructs), and known-groups validity (ability to distinguish between different phases of mobility recovery)^24,25^.

## Methods

### Study design

This cross-sectional analysis is part of the Mobilise-D clinical validation study (CVS; ISRCTN: 12051706), a multicentre prospective cohort study funded by the Innovative Medicines Initiative, aiming to clinically validate multiple DMOs in four clinical cohorts: PD, MS, COPD, and PFF^13^. Data were collected between April 2021 and June 2024. For this study, data from the first study visit of patients after PFF was used, based on the study database version 7.1.

### Ethics approval

This study was performed in line with the principles of the Declaration of Helsinki. Approval was granted at each study site by the Committee of the Protection of Persons, South-Mediterranean II, Montpellier (ref.: 221BO8), the EC of the Medical Faculty of Eberhard-Karls-University Tubingen, Stuttgart (ref.: 976/2020BO2), the EC of the Medical Faculty at Heidelberg University (ref.: S-719/2021), and the Regional Committee for Medical and Health Research Ethics, Central Norway (ref.: 216069).

### Participants

Participants were invited to an initial screening appointment. They were recruited through inpatient and outpatient lists across five sites in three European countries: Trondheim and Oslo (Norway), Stuttgart and Heidelberg (Germany), and Montpellier (France). Inclusion criteria required participants to be aged 45 years or older, be community-dwelling prior to the fracture, and have undergone surgical treatment (osteosynthesis or arthroplasty) for PFF. Participants were enrolled up to 52 weeks postoperatively. They had to be able to walk at least four metres at the first visit and be willing to wear a single wearable device. Further inclusion and exclusion criteria are listed in the study protocol^13^. Written informed consent was obtained from all participants included in the study^13^.

### Digital mobility outcomes

Real-world mobility performance of patients after PFFs was assessed for up to 7 days using one of two single wearable devices: the MoveMonitor+ worn on a belt around the waist (McRoberts B.V., The Hague, The Netherlands) or an Axivity AX6 (Axivity Ltd, Newcastle Upon Tyne, UK) fixed to the skin of the participants’ lower back with adhesive tape. Both devices included a 6 Degrees of Freedom inertial measurement unit with the following configuration: triaxial accelerometer with a range of +/-8 g and a resolution of 1 mg, triaxial gyroscope with a range of +/- 2000 degrees per second (deg/s) and a resolution of 70 milli-degrees per second (mili-deg/s), sampling frequency of 100 Hz (Hz). The devices were evaluated in a comprehensive technical validation study^26^. Both devices showed comparable technical specifications and met the predefined minimum performance requirements, ensuring no meaningful impact on algorithm performance.

We removed participants who did not have a minimum of at least 12 h of daily wear time (07:00–22:00 h) across ≥ 3 days^27^. In the preceding technical validation work, a broad array of available algorithms to detect digital mobility outcomes (DMOs) from a single wearable device were implemented according to agreed definitions^28^, and tested for criterion validity against gold and silver standards^19,20^. We first identified walking bouts (WBs, walking events of at least two strides of both feet^28^, and obtained WB-level DMOs aggregated at the weekly level^29^, resulting in 24 technically valid DMOs that encompass both walking activity (amount and pattern) and gait domains (pace, rhythm, and bout-to-bout variability)^19,20^. All 24 DMOs are described in Supplementary Table S1.

### Clinical outcome assessments (COAs)

We collected information on the following COAs: (i) Lower limb physical capacity measured using the Short Physical Performance Battery (SPPB), consisting of standing balance, gait speed, and five chair-rise tests, with total score as the outcome variable^30^; (ii) Mobility function and functional independence measured using the Timed Up and Go test (TUG), considering the total time taken to complete the task^31^; Walking ability as measured using (iii) the 4 m supervised gait speed test and (iv) the 6-minute walk test (6MinWT), including the endpoint of distance walked^32^. COAs were used for convergent validity.

### Patient-reported outcome measures (PROMs)

PROMs were used for convergent validity and included (i) perceived function using the function component of the Late-Life Function and Disability Instrument Functional Component (LLFDI-FC)^33^, (ii) concerns about falling using the Short Falls Efficacy Scale International (Short FES-I)^34^, and (iii) symptoms of fatigue measured using the Functional Assessment of Chronic Illness Therapy Fatigue Scale (FACIT-F)^35^.

### Other clinical outcomes

To test divergent validity, we collected information on: (i) systolic blood pressure; and (ii) hearing impairment during (a) a phone call and (b) a group conversation, assessed using the sum score of these two survey questions (range: 2–8), where lower scores indicate better hearing. These variables were a priori selected by a large expert panel in the Mobilise-D validation study.

### Statistical analysis

Assuming an alpha level of 0.05 and a power of 80%, we estimated that detecting statistically significant correlations of ≥ 0.6, ≥ 0.4, ≥ 0.3 and ≥ 0.2 would require sample sizes of 20, 46, 85, and 193 participants, respectively, based on Fisher’s z-transformation^36^. Given our available sample size, we had sufficient power to conduct the planned analyses, except for one subgroup as detailed below.

Statistical analyses were pre-specified and conducted using R Statistical Software (v4.3.2: R Core Team 2023). Given the absence of missing values in the constructs analysed in relation to the DMOs, and considering that missing DMO values were structurally determined by the absence of the corresponding walking behaviour, analyses were conducted using a complete-case approach. Data were tested for normality using histograms, QQ plots and skewness analysis. Patients’ characteristics and DMOs were described using mean (SD) or median (p25-p75), depending on distribution. This study followed the COSMIN guidelines for assessing construct validity, defining a priori hypotheses for both convergent and divergent validity, and including a rigorous assessment of known-groups validity^23^.

The convergent and divergent validity analyses were stratified a priori into an acute group (≤ 14 days since surgery) and a non-acute group (≥ 15 days after surgery). The acute group was kept separate for several reasons: (i) The Mobilise-D technical validation study identified challenges related to the accuracy and reliability of stride length and walking speed DMOs in patients after PFF with low physical capacity^21^; (ii) acute patients have very low physical capacity compared to patients in later recovery phases^8^; (iii) patients were not allowed to perform the 6MinWT and TUG tests during the acute state; and (iv) the LLFDI could not be collected meaningfully in the acute phase. The 14-day cut-off was selected as it reflects the transition from inpatient care to non-supervised independent mobility^37,38^.

To assess convergent validity, Pearson Product-Moment or Spearman Rank Correlation coefficients (with 95% CIs) were calculated between the 24 DMOs and the corresponding constructs (COAs and PROMs). A DMO was considered to demonstrate convergent validity when its correlation coefficients matched the hypothesised values^39^. The expected correlation coefficients are detailed in Supplementary Tables S2 and S3, and were based on a pilot study^20^, previous literature, and expert consultation. We expected no correlation to exceed 0.9, as that would indicate that the DMO provides the same information as existing constructs^39^. Correlation coefficients were classified as either very weak (< 0.3), weak (0.3–0.4), moderate (0.4–0.6), or strong (> 0.6), similar to previously proposed guidelines^40^. As some DMOs (e.g., bout-to-bout variability and rhythm domain) have not been measured under real-life conditions in patients after a PFF before, this part of the study was exploratory in nature. We hypothesised that the following would show weak to strong positive correlations with better physical capacity (COAs: high SPPB scores, fast gait speed, shorter TUG time) and mobility perception (PROMs: high LLFDI scores, low Short FES-I scores, high FACIT-F scores): (a) a higher number of steps and longer walking durations (amount), (b) a higher number of different WBs and WB durations (pattern), (c) faster walking speed (pace), (d) higher cadence and shorter stride duration (rhythm), and (e) greater bout-to-bout variability.

Divergent validity was assessed by analysing the Pearson Product-Moment or Spearman Rank Correlation coefficients between each of the DMOs and hearing loss and systolic blood pressure. The latter were expected to correlate very weakly with the DMOs (r/ρ’s < 0.2). Divergent validity was tested for the non-acute group only, as the sample size was insufficient for the acute group.

Known-groups validity was assessed by testing the distribution of DMOs across four PFF groups defined by time since surgery: acute (≤ 14 days), post-acute (15–42 days), extended recovery (43–182 days), and long-term recovery (≥ 183 days), consistent with established postoperative recovery trajectories^22^. These phases reflect clinically distinct stages of recovery, during which mobility is expected to improve progressively and then stabilise. Known-groups validity was supported when the p-for-trend from linear regression was ≤ 0.05. We expected statistically significant differences in mobility between the phases.

Final construct validity was determined through a structured expert consensus process. Nine experts from different professional backgrounds (geriatrics, orthopaedic surgery, physiotherapy, movement and exercise science, epidemiology, and data science) independently evaluated each DMO. Experts were provided with standardised summaries of the convergent, divergent and known-groups validity results for each DMO. They were instructed to base their judgment on the predefined assumptions described above and their clinical or methodological expertise. For each DMO, experts recorded a binary judgment (agree/disagree) on whether construct validity was achieved. These initial judgements were performed independently. Subsequently, disagreements were discussed in a joint meeting, during which experts could identify the reasoning behind differing opinions and clarify interpretations. Sources of discrepancy were documented. Final decisions on whether a DMO demonstrated construct validity were based on consensus.

## Results

From a total of 567 recruited participants after a PFF, the current analyses included 505 participants (89%), of which 100 (20%) were assessed in the acute phase, 117 (23%) in the post-acute phase, 201 (40%) in the extended recovery phase, and 87 (17%) in the long-term recovery phase (Table 1). No differences were observed between included and excluded participants, apart from higher indoor walking aid usage, less hearing impairment and a larger proportion of participants in the post-acute and extended recovery phases in the included sample (Supplementary Table S4). The reasons for exclusion were not using the wearable device (*n* = 24), not having available DMO measures due to technical problems (*n* = 11), or not having valid DMOs (*n* = 5 due to not having wear time available for technical reasons, and *n* = 22 not meeting the minimal wear time criteria).

**Table 1:** Sociodemographic characteristics for included participants after a PFF combined and grouped by four recovery phases.

	Total *N* = 505	Acute *n* = 100 (20%)	Post-acute *n* = 117 (23%)	Extended Recovery *n* = 201 (40%)	Long-term Recovery *n* = 87 (17%)
Age, mean (SD)	77.6 (9.4)	78.5 (8.5)	79.5 (9.2)	75.7 (9.7)	78 (9.5)
Sex, female, n (%)	333 (66%)	73 (73%)	74 (63%)	128 (64%)	58 (67%)
Marital status, married, n (%)	246 (49%)	48 (48%)	53 (45%)	106 (53%)	39 (45%)
Education less than high school (< 10 yrs) / others, n (%)	320 (63%)	60 (60%)	75 (64%)	125 (62%)	60 (69%)
Recruitment site, n (%)					
*Montpellier*	39 (8%)	3 (3%)	1 (1%)	21 (10%)	14 (16%)
*Trondheim / Oslo*	238 (47%)	92 (92%)	31 (26%)	83 (41%)	32 (37%)
*Stuttgart / Heidelberg*	228 (45%)	5 (5%)	85 (73%)	97 (48%)	41 (47%)
Fracture Type, n (%)					
*Femoral neck fracture*	348 (69%)	85 (85%)	74 (63%)	131 (65%)	58 (67%)
*Extracapsular fracture*	157 (31%)	15 (15%)	43 (37%)	70 (35%)	29 (33%)
Days since surgery, median (p25–p75)	60 (23–140)	3 (3–5)	28 (23–35)	98 (65–128)	325 (234–352)
Functional Comorbidity Index [0–18], mean (SD)	3.3 (2.1)	3.2 (2.1)	3.2 (2.1)	3.3 (2)	3.5 (2.3)
Walking Aids, n (%)					
*Indoor*	253 (62%)	-	25 (25%)	107 (91%)	115 (57%)
*Outdoor*	301 (74%)	-	42 (42%)	110 (94%)	143 (71%)

Acute patients were not included in the walking aid analysis as pre-fracture walking aid usage was assessed.

The included sample had more females (66%) than males, a mean (SD) age of 77.6 (9.4) years, and was measured at a median (p25-p75) of 60 (23–140) days after PFF surgery (Table 1). The full study sample had a mean (SD) SPPB score of 6.2 (3.1), a four-meter gait speed of 0.70 (0.40) m/s, a TUG time of 19.8 (12.3) s, and a 6MinWT distance of 283.6 (125.9) m (Table 2). Groups assessed early after surgery (acute and post-acute) showed worse physical function compared to those assessed in later recovery phases (extended and long-term) (Table 2). Acute patients walked a median of 519 (241–1069) steps in a total walking time of 7 (3–14) minutes per day (Supplementary Table S5), and non-acute participants walked a median of 2702 (1346–5280) steps with a total walking time of 32 (16–58) minutes per day (Table 3).

**Table 2:** Clinical characteristics for included participants after a PFF combined and grouped by four recovery phases.

	Total *N* = 505	Acute *n* = 100 (20%)	Post-acute *n* = 117 (23%)	Extended Recovery *n* = 201 (40%)	Long-term Recovery *n* = 87 (17%)
TUG (s), mean (SD)	19.8 (12.3)	-	25.5 (15.7)	17.5 (9)	17.4 (11.6)
SPPB					
*Total score [0–12]*,* mean (SD)*	6.2 (3.1)	3.5 (2.0)	5.5 (2.6)	7.3 (2.8)	7.6 (3.3)
*Gait speed 4MWT (m/s)*,* mean (SD)*	0.70 (0.40)	0.36 (0.20)	0.63 (0.22)	0.79 (0.34)	0.88 (0.38)
6MinWT Distance (m), mean (SD)	283.6 (125.9)	-	225.8 (104.5)	301.6 (124.4)	317.5 (131.3)
LLFDI-FC [0-100], median (p25-p75)	49 (42–59)	-	43 (40–49)	51 (45–58)	56 (46–65)
Short FES-I [7–28], median (p25-p75)	10 (7–14)	10 (7–15)	11 (8–15)	9 (7–13)	9 (7–12)
FACIT-F [0–52], median (p25-p75)	40 (31–45)	36 (29–42)	39 (30–44)	41 (34–46)	42 (33–46)
Hearing Impairment [2–8], mean (SD)	2.9 (1.2)	3.1 (1.2)	2.8 (1.1)	2.9 (1.2)	3.1 (1.2)
Systolic Blood Pressure (mm Hg), median (p25-p75)	132 (120–150)	130 (120–153)	130 (120–142)	135 (122–151)	136 (123–160)

*TUG* Timed Up and Go test; *SPPB* Short Physical Performance Battery; *4MWT* 4-Meter Walk Test; *6MinWT* 6-Minute Walking Test; *LLFDI-FC* Late Life Function and Disability Index – Functional Component; *Short FES-I* Short Falls Efficacy Scale International; *FACIT-F* Functional Assessment of Chronic Illness Therapy Fatigue Scale; *TUG*,* 5-time chair rise as part of SPPB*,* 6MinWT and LLFDI* Not performed in acute patients.

**Table 3:** Distribution and convergent and divergent validity (correlation coefficients with 95% CIs) of DMOs in 405 non-acute patients assessed ≥ 15 days after PFF surgery. Coefficients in bold represent those that matched or exceeded expected values, while coefficients in non-bold represent those that did not match the expected values.

DMO	Mean (SD) or median (p25 - p75)	Convergent							Divergent	
		TUG	SPPB total	4 m gait speed	6MinWT Distance	LLFDI-FC	Short FES-I	FACIT-F	Hearing Impairment	Systolic BP
Walking activity										
*Amount*										
Walking duration (min/day)	32 (16–58)	**-0.74 (-0.79**,** -0.69)**	**0.70 (0.64**,** 0.75)**	**0.68 (0.61**,** 0.73)**	**0.76 (0.70**,** 0.80)**	**0.71 (0.65**,** 0.75)**	**-0.43 (-0.51**,** -0.34)**	**0.38 (0.30**,** 0.46)**	**-0.13 (-0.22**,** -0.03)**	**0.01 (-0.09**,** 0.11)**
WB step count (steps/day)	2702 (1346–5280)	**-0.75 (-0.80**,** -0.70)**	**0.71 (0.66**,** 0.77)**	**0.70 (0.64**,** 0.75)**	**0.76 (0.71**,** 0.80)**	**0.72 (0.66**,** 0.76)**	**-0.43 (-0.51**,** -0.35)**	**0.38 (0.28**,** 0.46)**	**-0.14 (-0.23**,** -0.04)**	**0.01 (-0.09**,** 0.11)**
*Pattern*										
Number of WBs (n)	188 (139)	**-0.75 (-0.79**,** -0.70)**	**0.70 (0.64**,** 0.76)**	**0.68 (0.61**,** 0.73)**	**0.74 (0.70**,** 0.79)**	**0.72 (0.66**,** 0.76)**	**-0.40 (-0.48**,** -0.31)**	**0.37 (0.29**,** 0.44)**	**-0.14 (-0.23**,** -0.06)**	**0.06 (-0.05**,** 0.14)**
Number of WBs > 10s (n)	50 (25–94)	**-0.73 (-0.77**,** -0.67)**	**0.68 (0.61**,** 0.73)**	**0.67 (0.60**,** 0.72)**	**0.72 (0.66**,** 0.77)**	**0.68 (0.62**,** 0.73)**	**-0.42 (-0.49**,** -0.32)**	**0.37 (0.29**,** 0.45)**	**-0.14 (-0.23**,** -0.06)**	**0.02 (-0.08**,** 0.11)**
Number of WBs > 30s (n)	8 (3–16)	-0.57 (-0.64, -0.49)	0.54 (0.45, 0.61)	0.57 (0.50, 0.64)	0.59 (0.53, 0.66)	**0.52 (0.44**,** 0.59)**	-0.35 (-0.44, -0.26)	0.29 (0.19, 0.38)	**-0.12 (-0.21**,** -0.02)**	**-0.09 (-0.19**,** 0.02)**
Number of WBs > 60s (n)	2 (1–5)	**-0.52 (-0.59**,** -0.44)**	**0.50 (0.41**,** 0.57)**	**0.51 (0.42**,** 0.58)**	**0.54 (0.47**,** 0.61)**	**0.48 (0.41**,** 0.57)**	-0.33 (-0.42, -0.24)	0.25 (0.14, 0.34)	**-0.08 (-0.18**,** 0.01)**	**-0.04 (-0.14**,** 0.07)**
WB duration (s)	7.5 (6.9–8.4)	-0.01 (-0.11, 0.10)	-0.03 (-0.13, 0.06)	0.04 (-0.06, 0.14)	-0.02 (-0.12, 0.09)	-0.04 (-0.14, 0.06)	-0.09 (-0.18, 0.01)	0.10 (0.00, 0.20)	**-0.03 (-0.14**,** 0.07)**	**-0.09 (-0.18**,** 0.01)**
P90 WB duration (s)	20.3 (17.2–27.2)	-0.08 (-0.18, 0.02)	0.08 (-0.03, 0.17)	0.16 (0.06, 0.25)	0.07 (-0.03, 0.17)	0.04 (-0.07, 0.14)	-0.12 (-0.22, -0.02)	0.09 (-0.01, 0.19)	**-0.02 (-0.11**,** 0.08)**	**-0.15 (-0.24**,** -0.04)**
WB duration bout to bout variability (%)	104 (77–145)	**-0.51 (-0.58**,** -0.43)**	**0.50 (0.42**,** 0.57)**	**0.50 (0.42**,** 0.57)**	**0.55 (0.46**,** 0.61)**	**0.49 (0.40**,** 0.56)**	**-0.35 (-0.43**,** -0.25)**	0.27 (0.17, 0.35)	**-0.08 (-0.19**,** 0.02)**	**-0.03 (-0.14**,** 0.07)**
**Gait**										
*Pace*										
Walking speed in shorter (10–30 s) WBs (m/s)	0.63 (0.11)	**-0.71 (-0.76**,** -0.66)**	**0.63 (0.57**,** 0.69)**	**0.63 (0.56**,** 0.69)**	**0.70 (0.64**,** 0.75)**	**0.67 (0.62**,** 0.73)**	**-0.43 (-0.51**,** -0.35)**	**0.38 (0.28**,** 0.46)**	-0.21 (-0.29, -0.11)	**0.07 (-0.03**,** 0.17)**
Walking speed in longer (> 30s) WBs (m/s)	0.71 (0.15)	**-0.77 (-0.81**,** -0.72)**	**0.70 (0.65**,** 0.75)**	**0.68 (0.62**,** 0.74)**	**0.77 (0.73**,** 0.81)**	**0.72 (0.66**,** 0.77)**	**-0.45 (-0.53**,** -0.36)**	**0.40 (0.32**,** 0.48)**	**-0.18 (-0.26**,** -0.08)**	**0.05 (-0.06**,** 0.15)**
P90 walking speed in WBs > 10s (m/s)	0.77 (0.17)	**-0.77 (-0.81**,** -0.72)**	**0.70 (0.65**,** 0.75)**	**0.69 (0.62**,** 0.74)**	**0.77 (0.72**,** 0.81)**	**0.74 (0.68**,** 0.78)**	**-0.48 (-0.55**,** -0.39)**	**0.44 (0.36**,** 0.51)**	-0.22 (-0.31, -0.12)	**0.05 (-0.06**,** 0.16)**
P90 walking speed in longer (> 30s) WBs (m/s)	0.80 (0.20)	**-0.79 (-0.82**,** -0.74)**	**0.73 (0.67**,** 0.77)**	**0.70 (0.65**,** 0.75)**	**0.80 (0.76**,** 0.84)**	**0.74 (0.68**,** 0.79)**	**-0.46 (-0.54**,** -0.38)**	**0.42 (0.34**,** 0.50)**	**-0.18 (-0.28**,** -0.09)**	**0.02 (-0.08**,** 0.13)**
Stride length in shorter (10–30 s) WBs (cm)	90 (12)	**-0.52 (-0.59**,** -0.44)**	**0.44 (0.36**,** 0.53)**	**0.46 (0.37**,** 0.54)**	**0.55 (0.46**,** 0.62)**	**0.50 (0.41**,** 0.58)**	**-0.34 (-0.42**,** -0.24)**	**0.34 (0.25**,** 0.43)**	**-0.16 (-0.26**,** -0.06)**	**0.07 (-0.03**,** 0.17)**
Stride length in longer (> 30s) WBs (cm)	99 (15)	**-0.65 (-0.71**,** -0.59)**	**0.59 (0.51**,** 0.65)**	**0.56 (0.48**,** 0.63)**	**0.67 (0.60**,** 0.72)**	**0.60 (0.53**,** 0.66)**	**-0.40 (-0.47**,** -0.30)**	**0.38 (0.30**,** 0.47)**	**-0.13 (-0.24**,** -0.04)**	**0.06 (-0.04**,** 0.16)**
*Rythm*										
Cadence in all WBs (steps/min)	86 (8)	**-0.50 (-0.57**,** -0.42)**	**0.47 (0.39**,** 0.54)**	**0.48 (0.40**,** 0.54)**	**0.46 (0.39**,** 0.54)**	**0.47 (0.39**,** 0.53)**	-0.27 (-0.35, -0.17)	0.19 (0.09, 0.28)	**-0.11 (-0.20**,** -0.02)**	**0.03 (-0.06**,** 0.13)**
Cadence in longer (> 30s) WBs (steps/min)	85.64 (10)	**-0.51 (-0.59**,** -0.44)**	**0.47 (0.38**,** 0.55)**	**0.50 (0.42**,** 0.58)**	**0.50 (0.41**,** 0.57)**	**0.50 (0.42**,** 0.57)**	**-0.30 (-0.38**,** -0.21)**	0.23 (0.13, 0.32)	**-0.14 (-0.23**,** -0.03)**	**0.02 (-0.08**,** 0.12)**
P90 cadence in longer (> 30s) WBs (steps/min)	92 (12)	**-0.60 (-0.65**,** -0.52)**	**0.56 (0.48**,** 0.63)**	**0.58 (0.52**,** 0.64)**	**0.59 (0.51**,** 0.65)**	**0.56 (0.48**,** 0.62)**	**-0.33 (-0.42**,** -0.23)**	0.26 (0.17, 0.35)	**-0.15 (-0.24**,** -0.04)**	**-0.03 (-0.13**,** 0.07)**
Stride duration in all WBs (s)	1.28 (0.11)	0.31 (0.21, 0.40)	-0.28 (-0.36, -0.19)	-0.28 (-0.36, -0.19)	-0.26 (-0.34, -0.17)	**-0.30 (-0.38**,** -0.21)**	0.18 (0.08, 0.28)	-0.10 (-0.19, -0.01)	**0.08 (-0.03**,** 0.17)**	**0.02 (-0.08**,** 0.13)**
Stride duration in longer (> 30s) WBs (s)	1.30 (0.15)	**0.41 (0.32**,** 0.49)**	-0.36 (-0.45, -0.26)	-0.39 (-0.47, -0.29)	-0.37 (-0.46, -0.28)	**-0.41 (-0.49**,** -0.32)**	0.23 (0.13, 0.32)	-0.16 (-0.24, -0.06)	**0.12 (0.01**,** 0.21)**	**-0.01 (-0.10**,** 0.09)**
*Bout-to-bout variability*										
Walking speed bout-to-bout variability in longer (> 30s) WBs (%)	12 (5)	**-0.55 (-0.62**,** -0.47)**	**0.52 (0.44**,** 0.60)**	**0.47 (0.39**,** 0.54)**	**0.58 (0.51**,** 0.65)**	**0.50 (0.42**,** 0.58)**	-0.28 (-0.38, -0.18)	**0.33 (0.22**,** 0.41)**	**-0.15 (-0.24**,** -0.05)**	**-0.05 (-0.14**,** 0.05)**
Stride length bout-to-bout variability in longer (> 30s) WBs (%)	9 (4)	**-0.52 (-0.58**,** -0.43)**	**0.49 (0.41**,** 0.58)**	**0.42 (0.34**,** 0.50)**	**0.51 (0.43**,** 0.58)**	**0.48 (0.40**,** 0.55)**	-0.28 (-0.38, -0.18)	0.29 (0.19, 0.38)	**-0.12 (-0.22**,** -0.01)**	**-0.01 (-0.13**,** 0.10)**
Cadence bout-to-bout variability (%)	12 (2)	0.17 (0.06, 0.27)	-0.10 (-0.20, 0.00)	-0.11 (-0.21, -0.02)	-0.05 (-0.16, 0.06)	-0.13 (-0.23, -0.04)	0.09 (-0.00, 0.19)	-0.12 (-0.20, -0.02)	**0.03 (-0.07**,** 0.13)**	**-0.11 (-0.20**,** -0.01)**
Stride duration bout-to-bout variability (%)	18 (3)	0.22 (0.12, 0.31)	-0.18 (-0.27, -0.08)	-0.24 (-0.33, -0.15)	-0.16 (-0.27, -0.08)	-0.18 (-0.27, -0.09)	0.12 (0.02, 0.21)	-0.14 (-0.22, -0.03)	**0.04 (-0.06**,** 0.15)**	**-0.06 (-0.15**,** 0.06)**

*DMO* Digital Mobility Outcome; *TUG* Timed Up and Go test; *SPPB* Short Physical Performance Battery; *4 m gait speed* Gait speed from the SPPB supervised 4-meter walk test; *6MinWT* 6-Minute Walking Test; *LLFDI-FC* Late Life Function and Disability Index – Functional Component; *Short FES-I* Short Falls Efficacy Scale International; *FACIT-F* Functional Assessment of Chronic Illness Therapy Fatigue Scale; *BP* Blood Pressure; *WBs* Walking Bouts.

Number of non-acute participants missing DMO values: Walking speed in shorter (10–30 s) WBs, P90 walking speed in WBs > 10s, Stride length in shorter (10–30 s) WBs: 1 missing value. Walking speed in longer (> 30s) WBs, P90 walking speed in longer (> 30s) WBs, Stride length in longer (> 30s) WBs, Cadence in longer (> 30s) WBs, P90 cadence in longer (> 30s) WBs, Stride duration in longer (> 30s) WBs: 10 missing values. Walking speed bout-to-bout variability in longer (> 30s) WBs, Stride length bout-to-bout variability in longer (> 30s) WBs: 22 missing values.

### Convergent validity

The walking amount domain DMOs showed very weak to strong correlations with the related constructs (|ρ| = 0.08–0.76) (non-acute group, *n* = 405: Table 3; acute group, *n* = 100: Supplementary Table S5), consistent with the anticipated coefficients (non-acute group: Supplementary Table S2; acute group: Supplementary Table S3). Similarly, walking pattern and pace DMOs showed very weak to strong correlations with related constructs (|ρ| = 0.01–0.80), with the vast majority matching the expected coefficients in the non-acute group. Rhythm DMOs provided very weak to strong correlations (|*ρ*| = 0.01–0.60), with three of five coefficients in the non-acute group matching the expected correlations. The bout-to-bout variability DMOs demonstrated very weak to moderate associations with the related constructs (|*ρ*| = 0.02–0.58), with less than half of the coefficients meeting the expected correlations. Overall, DMOs in the non-acute group demonstrated stronger and more consistent correlations with related constructs compared to the acute group, with a larger number of coefficients aligning with expected values. Also, correlations between DMOs and COAs were consistently stronger than between DMOs and PROMs, matching the expectations.

### Divergent validity

Very weak correlations were observed between all DMOs and the constructs of divergent validity (|*ρ*| = 0.01–0.22) for the non-acute group (Table 3), as expected (Supplementary Table S2). Only two DMOs exceeded the expected correlation threshold of 0.2, namely walking speed in shorter WBs (*ρ* = -0.21) and P90 walking speed in WBs > 10s (*ρ* = -0.22).

### Known-groups validity

In total, 21 of 24 DMOs statistically differentiated across the four recovery groups (Supplementary Fig. S6), supporting known-groups validity. The three DMOs that did not differentiate between the recovery groups were WB duration, P90 WB duration, and stride length bout-to-bout variability in longer (> 30s) WBs.

### Expert consensus on construct validity

There was expert consensus that 17 of the 24 DMOs showed evidence for construct validity, covering almost all DMOs in the domains of amount (2 of 2), pattern (5 of 7), and pace (6 of 6), and fewer DMOs in the domains of rhythm (3 of 5) and bout-to-bout variability (1 of 4). Experts considered the three cadence DMOs and the walking speed bout-to-bout variability in longer (> 30s) WBs as valid but exploratory. The independent voting showed that among the 17 DMOs considered valid, three had unanimous expert agreement (100% in favour) and 14 had minor disagreements (78–89% in favour) (Table 4). All disagreements were discussed in the subsequent joint expert meeting, after which a consensus decision was made on all the DMOs (Table 4). Experts placed larger emphasis on the results for the non-acute group and gave greater weight to correlation coefficients with COAs than PROMs. There was expert consensus that 7 DMOs did not show evidence for construct validity: two pattern DMOs (mean and P90 WB duration), two rhythm DMOs (stride duration in all WBs and in longer (> 30s) WBs), and three bout-to-bout variability DMOs (stride length bout to bout variability in longer (> 30s) WBs, cadence bout-to-bout variability, and stride duration bout-to-bout variability).

**Table 4:**
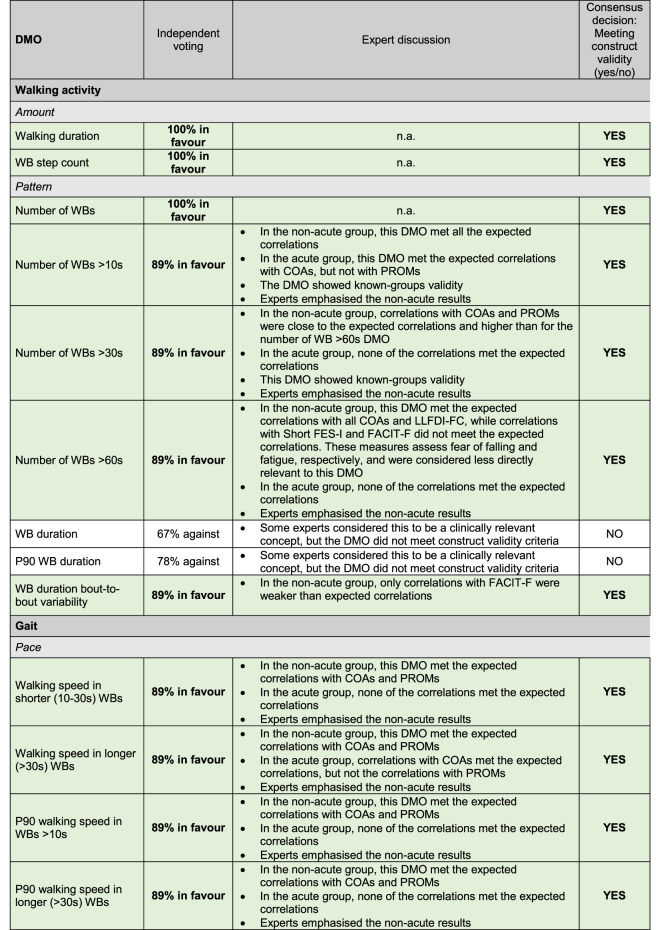

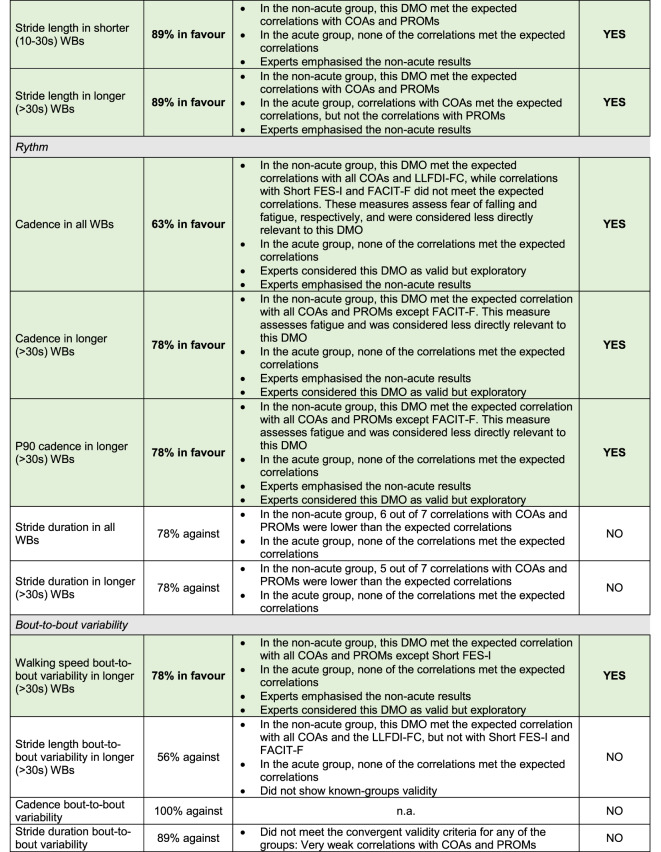
Independent voting, expert discussion, and final consensus decision about whether each DMO met construct validity or not. Bold text and green highlight indicate that construct validity was met.

*WBs* Walking Bouts; *n.a.* not applicable; *DMO* Digital Mobility Outcome; *PROMs* Patient-Reported Outcome Measures; *COAs* Clinical Outcome Assessments; *LLFDI-FC* Late Life Function and Disability Index – Functional Component; *Short FES-I* Short Falls Efficacy Scale International; *FACIT-F* Functional Assessment of Chronic Illness Therapy Fatigue Scale.

## Discussion

This is the first multi-site study to evaluate the construct validity of real-world DMOs in a large representative sample of both acute and non-acute patients within the first year of recovery following PFF - a population at high risk of poor mobility recovery, loss of functional independence, care home admission, and accelerated mortality. Based on consensus and focus on non-acute patients, evidence for construct validity was observed for 17 of 24 DMOs, specifically in (almost) all DMOs of amount (2 of 2), pattern (5 of 7), and pace (6 of 6) domains. Evidence for construct validity was more variable for the rhythm (3 of 5) and bout-to-bout variability (1 of 4) domains. Accordingly, the majority of DMOs can capture the intended mobility and health aspects in patients after a PFF, providing valid insights into their health status, complementing clinical tests in non-acute and home-based settings.

### Amount of walking activity

Our results demonstrate construct validity for the DMOs WB step count and walking duration, supported by our convergent, divergent and known-groups validity analyses. The consistent strong correlations between DMOs and related mobility constructs even exceeded correlation coefficients found in previous observational studies^17,41,42^. The WB step count and walking duration have the discriminative power to distinguish between patients in different recovery phases. However, the differences are particularly accentuated between the earlier phases. This aligns with previous findings of longitudinal observational studies, which observed a steep increase in mobility capacity in the first six months and subsequent stabilisation in months 6–12^6–8^, with the number of steps per WB peaking nine months postoperatively^9^.

### Pattern of walking activity

Seven walking activity pattern DMOs were assessed, of which five demonstrated construct validity, namely the number of WBs per day in the different WB duration categories (overall, and longer than 10s, 30s, 60s) and the variability in WB duration. These DMOs showed moderate to strong correlations with the COAs, of which four met the predefined thresholds for convergent validity. Although the coefficients for the number of WBs > 30s remained slightly below the a priori threshold of ρ = 0.6, the expert panel regarded this DMO as valid due to its consistent moderate associations with related mobility constructs and its support from both divergent and known-groups validity.

Unexpectedly, the two DMOs representing WB duration did not meet the criteria for construct validity. A likely explanation is that most WBs were under 10s across all recovery phases, with a median WB duration in our study of 7.5 s. Such short durations may be feasible for most patients, regardless of their physical capacity. Previous studies have similarly reported a large number of short WBs during the entire recovery process after a PFF^18^. The latter study also highlighted the potential environmental impact on WB duration, as a decline was observed in patients’ WB duration from the hospital to the home environment^18^. People’s home environment may offer limited space for longer WBs, in contrast to hospitals with long corridors. While very short WBs are frequent and clinically relevant in patients after a PFF^18,21^, their ability to represent typical walking patterns is questionable, since they often occur indoors in cluttered spaces and may include other daily activities^43^.

### Pace of gait

Real-world walking speed and stride length are two prominent gait metrics whose construct validity has not yet been established in people after a PFF. In this study, all pace-related DMOs met the expected moderate to strong correlations with the related constructs in the non-acute group, with most of them showing divergent validity as well. In the acute group, weak to moderate expected correlations were observed only for walking speed and stride length in longer WBs. The absence of associations in shorter WBs may be attributed to overestimation of these DMO estimates in individuals with lower physical capacity and slower walking speed after a PFF, leading to reduced variability and a more homogeneous sample^19,21^. This finding highlights a broader methodological challenge: short WB durations in combination with severe mobility limitations pose difficulties for the accurate estimation of real-world walking speed by sensor-based algorithms^19,21^. Therefore, only WBs longer than 10 s were included in the clinical validation of pace-related DMOs. Although convergent validity was not consistently demonstrated in the acute group, known-groups validity was observed across all four recovery phases, and the expert group reached consensus that all pace DMOs fulfilled construct validity for the non-acute group. These findings are of high clinical relevance, as supervised walking speed has high predictive capacity for long-term functional changes and mortality risk in older adults^44,45^.

### Rhythm of gait

Cadence and stride duration represent the temporal and rhythmic components of gait. Despite all five rhythm DMOs differentiating between the four phases of PFF recovery (Supplementary Fig. S6), only the three cadence DMOs showed evidence of construct validity. This may be attributed to the higher accuracy and reliability of cadence estimation compared to stride duration estimation in PFF^21^. The present results indicated that higher cadence was moderately associated with better mobility capacity, whereas step duration showed weaker associations. This finding is somewhat surprising, as a previous study conducted in laboratory-based conditions demonstrated high intercorrelations between cadence and step duration, with both closely linked to metrics of pace^46^. Given the inconsistent associations between rhythm DMOs and measures of mobility capacity, in combination with the lack of evidence in real-world settings, our clinical experts considered the domain of rhythm to remain exploratory and recommend further investigation to gain insight into the clinical meaning of cadence after a PFF. Additional rhythm-related DMOs – such as measures of gait asymmetry (e.g., right-left imbalance) – may offer further insight into recovery trajectories and should be explored in future follow-up assessments of patients after PFF^46^.

### Bout-to-bout variability of gait

The bout-to-bout variability domain captures fluctuations in walking behaviour – such as in stride duration, stride length, speed, or cadence – across separate walking episodes throughout the day. This aspect of mobility has not been investigated before in individuals recovering from PFF. Of the four variability-related DMOs, only bout-to-bout variability of walking speed in longer WBs correlated moderately with mobility capacity in the non-acute group and distinguished between recovery phases. This finding suggests that individuals with greater mobility capacity may display higher variability in real-world walking speed. In this context, higher variability between WBs may indicate adaptivity to varying environmental demands, as previously described in individuals with Parkinson’s disease^43^. From a clinical perspective, bout-to-bout variability could complement traditional average-based mobility metrics by capturing flexibility and context-dependent modulation of real-world walking.

The remaining three bout-to-bout variability DMOs (in stride duration, stride length, and cadence) failed to meet the criteria for construct validity. Stride length bout-to-bout variability showed a moderate association with mobility capacity, but did not show known-groups validity, possibly due to the technical challenges in estimating stride length in PFF^21^. In sum, walking speed bout-to-bout variability in longer bouts may be a relevant DMO in non-acute patients. However, further research is needed to better understand its potential clinical relevance and utility in monitoring patients after a PFF.

### Construct validity of DMOs in the acute phase after PFF

As anticipated, correlations between DMOs and established clinical outcome measures were substantially lower in acute patients compared to non-acute patients. Notably, only six DMOs – spanning the domains of amount, pattern, and pace - met the expected correlations with COAs. The weak to moderate correlation coefficients observed between the amount-related DMOs and COAs align with previous research that reported moderate correlations between upright time and TUG in a small sample of acute patients^16^. However, direct comparison is limited due to differences in DMO definitions and methodologies across studies.

The low associations observed in the acute group may reflect the severely reduced mobility typically seen in the early stages of recovery after PFF surgery^16,47,48^. During this phase, patients often show restricted physical function and reduced ability to walk for longer durations^22^, resulting in fewer WBs of sufficient duration for reliable DMO estimations. Moreover, the acute sample tends to be relatively homogenous regarding mobility behaviour and capacity, which limits variation and hence statistical power. Additionally, walking behaviour in this setting is likely influenced more by structured clinical routines than by real-world autonomy.

### Implications

Our findings support the use of DMOs for monitoring and guiding the care of older adults in the year following PFF surgery, although DMOs may have limited utility during the first two weeks post-surgery and should be supplemented with more traditional clinical tests of physical capacity in this acute phase. Clinically validated real-world DMOs derived from a single wearable device offer promising opportunities for remote monitoring of home-dwelling older adults after PFF. First, mobility monitoring might serve in the prediction of fall risk^49^ and poor mobility recovery, which are currently being examined within the Mobilise-D consortium. Second, validated DMOs could facilitate the design and implementation of personalised rehabilitation programs^50^, with tailored exercise interventions informed by patients’ functional capacity and environmental constraints. Objective mobility data may also serve as motivational feedback, potentially enhancing engagement and the effectiveness of transitional care following PFF^51^. Third, remote monitoring offers logistical and economic advantages. Its non-intrusive nature can increase adherence to long-term monitoring by eliminating the need for frequent clinical visits – an especially important factor for individuals in rural or underserved areas^52^. The feasibility of this approach is underscored by the high completion rate of > 90%, defined as wear time of at least three days and 12 h per day. Reducing the burden of travel and the time demands on clinical staff may also contribute to cost savings, while enabling continuous and objective assessment of recovery in real-world settings.

### Strengths and limitations

This study included a large and diverse sample of patients recovering from PFF, recruited across three countries at various time points up to one year post-surgery. This broad recruitment strategy enhances the generalizability of findings by including patients at various stages of functional recovery and from different healthcare settings. A further strength lies in the application of a comprehensive construct validity framework following the COSMIN guidelines, assessing convergent, divergent, and known-groups validity^23^. Notably, the known-groups approach based on recovery phase has not previously been applied in studies validating DMOs in patients after hip fracture. The DMOs’ ability to differentiate between these recovery phases supports the validity of this approach. Furthermore, this study followed a rigorous protocol for data quality with predefined thresholds for valid wear time, ensuring high data reliability. The validity of the findings was strengthened by the use of a priori hypotheses and multi-professional expert consensus, aligning with best practices for digital biomarker validation. However, some limitations must be acknowledged. Key clinical assessments relevant to convergent validity, such as the SPPB, TUG and LLFDI, could not be performed in the acute recovery phase. Additionally, differences in healthcare systems across countries may have influenced recovery trajectories and mobility outcomes. For example, acute participants in Germany typically receive more intensive and prolonged inpatient rehabilitation, whereas participants in Norway are often discharged to home at an earlier stage. The number of patients in the acute group was too small to allow for site-specific analyses or to examine divergent validity.

In addition to validating the DMOs against established clinical tests of mobility capacity, PROMs were also included as criteria for convergent validity. This reflects Mobilise-D’s holistic view of mobility, which encompasses capacity, daily performance, and patients’ perceptions^14^. Another strength is the inclusion of DMOs across multiple mobility domains. However, this introduces some methodological challenges. Among the PROMs included, the Short FES-I and the FACIT-F primarily assess fear of falling and fatigue, respectively, which are related to mobility but do not directly capture mobility performance. This may partly explain their weaker associations with the DMOs compared to measures that more explicitly reflect functional mobility. For domains such as rhythm and bout-to-bout variability, where limited prior evidence exists, clinical expertise was required to develop a priori hypotheses. Consequently, these aspects of the study are exploratory in nature. Further research is needed to improve the understanding of the interpretability and clinical relevance of DMOs related to rhythm and bout-to-bout variability, including a possible expansion of these domains.

### Conclusion

A total of 17 DMOs related to walking activity and gait demonstrated evidence supporting construct validity in non-acute patients recovering from PFF. Construct validity was supported through rigorous statistical testing and expert consensus, incorporating convergent, divergent, and known-groups validity in accordance with the COSMIN guidelines. Specifically, all DMOs within the amount and pace domains, as well as most within the pattern domain, demonstrated construct validity, whereas a minority of rhythm and bout-to-bout variability DMOs fulfilled these criteria. In sum, real-world DMOs offer a cost-effective and clinically relevant solution with strong clinimetric properties, capable of reflecting a diverse range of clinical outcomes – from objective functional mobility capacity to patients’ subjective perceptions about their mobility. These DMOs may deepen our understanding of functional recovery outside controlled clinical settings, offering clinicians valuable information about how functional gains translate into daily life. In addition, evidence for construct validity is a necessary but not sufficient step towards regulatory endorsement of DMOs in for example PFF rehabilitation or routine follow-up. Additional evidence on responsiveness, predictive validity, longitudinal outcomes, and clinically meaningful change is needed before regulatory endorsement can be supported.

## Supplementary Information

Below is the link to the electronic supplementary material.

Supplementary Material 1

## Data Availability

The data presented in this paper was generated during the IMI2 Mobilise-D project. The dataset from this project will be released under a C.C.4.0 NC license in June 2026 at https://zenodo.org/communities/mobilise-d/ according to the data release schedule agreed by the Mobilise-D consortium. Please direct any data-related queries to: Brian CaulfieldEmail: b.caulfield@ucd.ie.
