# Supplementary material for: Construct validity of real-world digital mobility outcomes in patients after proximal femoral fracture: a cross-sectional observational study
Source: Sci Rep. 2026 Mar 20;16:9535. doi: 10.1038/s41598-026-43297-y (PMC13005024; doi:10.1038/s41598-026-43297-y)
Supplement: Supplementary file 1 — Supplementary Material 1 [file 41598_2026_43297_MOESM1_ESM.docx]

# Supplementary Information:

Construct validity of real-world digital mobility outcomes in patients after proximal femoral fracture: A cross-sectional observational study

Tobias Eckert*^1,2^, Martin Aursand Berge*^3^, Michael Long^4^, Marti de las Heras^5,6,7^, Paula Alvarez^5^, Hubert Blain^8^, Julia Braun^9^, Joren Buekers^5,6,7^, Brian Caulfield^10,11^, Monika Engdal^3^, Anja Frei^9^, Jorunn L. Helbostad^3^, Anisoara Ionescu^12^, Carl-Philipp Jansen^1,2^, Lars Gunnar Johnsen^3,13^, Jochen Klenk^1,14,15^, Sarah Koch ^5,6,7,16^, Vita Lanfranchi^4^, Lynn Rochester^17,18^, Clemens Becker^1^, Beatrix Vereijken^3^, Judith Garcia-Aymerich*^5,6,7^

1. Robert Bosch Foundation for Medical Research, Stuttgart, Germany
2. Geriatric Center, Medical Faculty Heidelberg, Heidelberg University, Heidelberg, Germany
3. Department of Neuromedicine and Movement Science, Norwegian University of Science and Technology, Trondheim, Norway
4. Department of Computer Science, University of Sheffield, Sheffield, UK
5. ISGlobal, Barcelona, Spain
6. Universitat Pompeu Fabra (UPF), Barcelona, Spain
7. CIBER Epidemiología y Salud Pública (CIBERESP), Barcelona, Spain.
8. University Hospital of Montpellier, Montpellier, France
9. Epidemiology, Biostatistics and Prevention Institute, University of Zurich, Switzerland
10. School of Public Health, Physiotherapy & Population Science, University College Dublin, D04 V1W8 Dublin, Ireland
11. Insight Research Ireland Centre For Data Analytics, University College Dublin, D04 P7W1 Dublin, Ireland
12. École Polytechnique Fédérale de Lausanne, Lausanne, Switzerland
13. Department of Orthopaedic Surgery, St. Olav’s Hospital, Trondheim, Norway
14. Institute of Epidemiology and Medical Biometry, Ulm University, Ulm, Germany
15. Study Center Stuttgart, IB University of Health and Social Sciences, Stuttgart, Germany
16. Department of Sport, Exercise, and Health, University of Basel, Switzerland
17. Translational and Clinical Research Institute, Newcastle University, Newcastle Upon Tyne NE4 5PL, United Kingdom
18. National Institute for Health and Care Research (NIHR), Newcastle Biomedical Research Centre (BRC), Newcastle University and The Newcastle upon Tyne Hospitals NHS Foundation Trust, Newcastle Upon Tyne NE4 5PL, United Kingdom

**Shared first authors:** Tobias Eckert, Martin Aursand Berge, Michael Long

* Corresponding authors

Primary corresponding author: Tobias Eckert

**Supplementary Table S1**. Description of the 24 digital mobility outcomes (DMOs).

| **DMO** | **Definition** | **Unit** |
| --- | --- | --- |
| **Walking activity** |  |  |
| *Amount* |  |  |
| Walking duration | Weekly mean of time spent walking per day | min/day |
| WB step count | Weekly mean of the number of steps per day | steps/day |
| *Pattern* |  |  |
| Number of WBs (n) | Weekly mean of the sum of walking bouts per day | WBs/day |
| Number of WBs >10s | Weekly mean of the sum of walking bouts per day including bouts longer than 10 seconds | WBs/day |
| Number of WBs >30s | Weekly mean of the sum of walking bouts per day including bouts longer than 30 seconds | WBs/day |
| Number of WBs >60s | Weekly mean of the sum of walking bouts per day including bouts longer than 60 seconds | WBs/day |
| WB duration | Weekly mean of the daily mean of walking bout duration | s |
| P90 WB duration | Weekly mean of the daily 90th percentile of walking bout duration | s |
| WB duration bout to bout variability | Weekly mean of the daily bout to bout variability of walking bout duration | % |
| **Gait** |  |  |
| *Pace* |  |  |
| Walking speed in shorter (10-30s) WBs | Weekly mean of the daily average walking speed, assessed in walking bouts between 10  and 30 seconds | m/s |
| Walking speed in longer (>30s) WB | Weekly mean of the daily average walking speed, assessed in walking bouts longer than 30 seconds | m/s |
| P90 walking speed in WBs >10s | Weekly mean of the daily 90th percentile of the walking speed, assessed in walking bouts of more than 10 seconds | m/s |
| P90 walking speed in longer (>30s) WBs | Weekly mean of the daily 90th percentile of the walking speed, assessed in walking bouts of longer than 30 seconds | m/s |
| Stride length in shorter (10-30s) WBs | Weekly mean of the daily length of two consecutive steps, assessed during walking  bouts between 10 and 30 seconds | cm |
| Stride length in longer (>30s) WBs | Weekly mean of the daily average length of two consecutive steps, assessed during walking bouts longer than 30 seconds | cm |
| *Rythm* |  |  |
| Cadence in all WBs | Weekly mean of the daily average of the steps frequency during a period of time (minutes), calculated in all walking bouts | steps/min |
| Cadence in longer (>30s) WBs | Weekly mean of the daily average of the steps frequency during a period of time (minutes), calculated in walking bouts longer than 30 seconds | steps/min |
| P90 cadence in longer (>30s) WBs | Weekly mean of the daily 90th percentile of the steps frequency during a period of time (minutes), calculated in walking bouts longer than 30 seconds | steps/min |
| Stride duration in all WBs | Weekly mean of the daily average of the time elapsed between the initial contacts of two  consecutive footfalls of the same foot, assessed in all walking bouts | s |
| Stride duration in longer (>30s) WBs | Weekly mean of the daily average of the time elapsed between the initial contacts of two  consecutive footfalls of the same foot, assessed in walking bouts longer than 30 seconds | s |
| *Bout-to-bout variability* |  |  |
| Walking speed bout-to-bout variability in longer (>30s) WBs | Weekly mean of the daily bout-to-bout variability of walking speed, assessed in walking  bouts longer than 30 seconds | % |
| Stride length bout-to-bout variability in longer (>30s) WBs | Weekly mean of the daily bout-to-bout variability of stride length, assessed in walking  bouts longer than 30 seconds | % |
| Cadence bout-to-bout variability | Weekly mean of the daily bout-to-bout variability of cadence, assessed in all walking  bouts | % |
| Stride duration bout-to-bout variability | Weekly mean of the daily bout-to-bout variability of stride duration, assessed in all  walking bouts | % |

**Supplementary Table S2**. Expected correlation coefficient ranges between DMOs and related constructs in **non-acute** patients, derived from previous research, pilot testing, and expert consultation.

| **DMO** |  |  |  | **Convergent** |  |  |  |
| --- | --- | --- | --- | --- | --- | --- | --- |
|  | **TUG** | **SPPB total** | **4m gait speed** | **6MinWT Distance** | **LLFDI-FC** | **Short FES-I** | **FACIT-F** |
| **Walking activity** |  |  |  |  |  |  |  |
| *Amount* |  |  |  |  |  |  |  |
| Walking duration (min/day) | -0.6 to -0.9 | 0.6 to 0.9 | 0.6 to 0.9 | 0.6 to 0.9 | 0.4 to 0.6 | -0.4 to -0.6 | 0.3 to 0.4 |
| WB step count (steps/day) | -0.6 to -0.9 | 0.6 to 0.9 | 0.6 to 0.9 | 0.6 to 0.9 | 0.4 to 0.6 | -0.4 to -0.6 | 0.3 to 0.4 |
| *Pattern* |  |  |  |  |  |  |  |
| Number of WBs (n) | -0.6 to -0.9 | 0.6 to 0.9 | 0.6 to 0.9 | 0.6 to 0.9 | 0.4 to 0.6 | -0.4 to -0.6 | 0.3 to 0.4 |
| Number of WBs >10s (n) | -0.6 to -0.9 | 0.6 to 0.9 | 0.6 to 0.9 | 0.6 to 0.9 | 0.4 to 0.6 | -0.4 to -0.6 | 0.3 to 0.4 |
| Number of WBs >30s (n) | -0.6 to -0.9 | 0.6 to 0.9 | 0.6 to 0.9 | 0.6 to 0.9 | 0.4 to 0.6 | -0.4 to -0.6 | 0.3 to 0.4 |
| Number of WBs >60s (n) | -0.4 to -0.6 | 0.4 to 0.6 | 0.4 to 0.6 | 0.6 to 0.9 | 0.4 to 0.6 | -0.4 to -0.6 | 0.3 to 0.4 |
| WB duration (s) | -0.4 to -0.6 | 0.4 to 0.6 | 0.4 to 0.6 | 0.4 to 0.6 | 0.4 to 0.6 | -0.4 to -0.6 | 0.3 to 0.4 |
| P90 WB duration (s) | -0.4 to -0.6 | 0.4 to 0.6 | 0.4 to 0.6 | 0.6 to 0.9 | 0.4 to 0.6 | -0.4 to -0.6 | 0.3 to 0.4 |
| WB duration bout to bout variability (%) | -0.4 to -0.6 | 0.4 to 0.6 | 0.4 to 0.6 | 0.4 to 0.6 | 0.4 to 0.6 | -0.4 to -0.6 | 0.3 to 0.4 |
| **Gait** |  |  |  |  |  |  |  |
| *Pace* |  |  |  |  |  |  |  |
| Walking speed in shorter (10-30s) WBs (m/s) | -0.4 to -0.6 | 0.4 to 0.6 | 0.4 to 0.6 | 0.4 to 0.6 | 0.4 to 0.6 | -0.3 to -0.4 | 0.3 to 0.4 |
| Walking speed in longer (>30s) WBs (m/s) | -0.4 to -0.6 | 0.4 to 0.6 | 0.4 to 0.6 | 0.4 to 0.6 | 0.4 to 0.6 | -0.3 to -0.4 | 0.3 to 0.4 |
| P90 walking speed in WBs >10s (m/s) | -0.6 to -0.9 | 0.6 to 0.9 | 0.6 to 0.9 | 0.6 to 0.9 | 0.4 to 0.6 | -0.4 to -0.6 | 0.3 to 0.4 |
| P90 walking speed in longer (>30s) WBs (m/s) | -0.6 to -0.9 | 0.6 to 0.9 | 0.6 to 0.9 | 0.6 to 0.9 | 0.4 to 0.6 | -0.4 to -0.6 | 0.3 to 0.4 |
| Stride length in shorter (10-30s) WBs (cm) | -0.4 to -0.6 | 0.4 to 0.6 | 0.4 to 0.6 | 0.4 to 0.6 | 0.4 to 0.6 | -0.3 to -0.4 | 0.3 to 0.4 |
| Stride length in longer (>30s) WBs (cm) | -0.4 to -0.6 | 0.4 to 0.6 | 0.4 to 0.6 | 0.4 to 0.6 | 0.4 to 0.6 | -0.3 to -0.4 | 0.3 to 0.4 |
| *Rythm* |  |  |  |  |  |  |  |
| Cadence in all WBs (steps/min) | -0.4 to -0.6 | 0.4 to 0.6 | 0.4 to 0.6 | 0.4 to 0.6 | 0.3 to 0.4 | -0.3 to -0.4 | 0.3 to 0.4 |
| Cadence in longer (>30s) WBs (steps/min) | -0.4 to -0.6 | 0.4 to 0.6 | 0.4 to 0.6 | 0.4 to 0.6 | 0.3 to 0.4 | -0.3 to -0.4 | 0.3 to 0.4 |
| P90 cadence in longer (>30s) WBs (steps/min) | -0.4 to -0.6 | 0.4 to 0.6 | 0.4 to 0.6 | 0.4 to 0.6 | 0.3 to 0.4 | -0.3 to -0.4 | 0.3 to 0.4 |
| Stride duration in all WBs (s) | 0.4 to 0.6 | -0.4 to -0.6 | -0.4 to -0.6 | -0.4 to -0.6 | -0.3 to -0.4 | 0.3 to 0.4 | -0.3 to -0.4 |
| Stride duration in longer (>30s) WBs (s) | 0.4 to 0.6 | -0.4 to -0.6 | -0.4 to -0.6 | -0.4 to -0.6 | -0.3 to -0.4 | 0.3 to 0.4 | -0.3 to -0.4 |
| *Bout-to-bout variability* |  |  |  |  |  |  |  |
| Walking speed bout-to-bout variability in longer (>30s) WBs (%) | -0.4 to -0.6 | 0.4 to 0.6 | 0.4 to 0.6 | 0.4 to 0.6 | 0.3 to 0.4 | -0.3 to -0.4 | 0.3 to 0.4 |
| Stride length bout-to-bout variability in longer (>30s) WBs (%) | -0.4 to -0.6 | 0.4 to 0.6 | 0.4 to 0.6 | 0.4 to 0.6 | 0.3 to 0.4 | -0.3 to -0.4 | 0.3 to 0.4 |
| Cadence bout-to-bout variability (%) | -0.4 to -0.6 | 0.4 to 0.6 | 0.4 to 0.6 | 0.4 to 0.6 | 0.3 to 0.4 | -0.3 to -0.4 | 0.3 to 0.4 |
| Stride duration bout-to-bout variability (%) | -0.4 to -0.6 | 0.4 to 0.6 | 0.4 to 0.6 | 0.4 to 0.6 | 0.3 to 0.4 | -0.3 to -0.4 | 0.3 to 0.4 |

*DMO* Digital Mobility Outcome; TUG Timed Up and Go test; SPPB Short Physical Performance Battery; 4m gait speed Gait speed from the SPPB supervised 4-meter walk test; 6MinWT 6-Minute Walking Test; *LLFDI-FC* Late Life Function and Disability Instrument – Functional Component; *Short FES-I* Short Falls Efficacy Scale International; *FACIT-F* Functional Assessment of Chronic Illness Therapy Fatigue Scale; *WBs* Walking Bouts.

**Supplementary Table S3**. Expected correlation coefficient ranges between DMOs and related constructs in **acute** patients, derived from previous research, pilot testing, and expert consultation.

| **DMO** |  | **Convergent** |  |  |
| --- | --- | --- | --- | --- |
|  | **SPPB total** | **4m gait speed** | **Short FES-I** | **FACIT-F** |
| **Walking activity** |  |  |  |  |
| *Amount* |  |  |  |  |
| Walking duration (min/day) | 0.3 to 0.4 | 0.3 to 0.4 | -0.3 to -0.4 | 0.3 to 0.4 |
| WB step count (steps/day) | 0.3 to 0.4 | 0.3 to 0.4 | -0.3 to -0.4 | 0.3 to 0.4 |
| *Pattern* |  |  |  |  |
| Number of WBs (n) | 0.3 to 0.4 | 0.3 to 0.4 | -0.3 to -0.4 | 0.3 to 0.4 |
| Number of WBs >10s (n) | 0.3 to 0.4 | 0.3 to 0.4 | -0.3 to -0.4 | 0.3 to 0.4 |
| Number of WBs >30s (n) | 0.3 to 0.4 | 0.3 to 0.4 | -0.3 to -0.4 | 0.3 to 0.4 |
| Number of WBs >60s (n) | 0.3 to 0.4 | 0.3 to 0.4 | -0.3 to -0.4 | 0.3 to 0.4 |
| WB duration (s) | 0.3 to 0.4 | 0.3 to 0.4 | -0.3 to -0.4 | 0.3 to 0.4 |
| P90 WB duration (s) | 0.3 to 0.4 | 0.3 to 0.4 | -0.3 to -0.4 | 0.3 to 0.4 |
| WB duration bout to bout variability (%) | 0.3 to 0.4 | 0.3 to 0.4 | -0.3 to -0.4 | 0.3 to 0.4 |
| **Gait** |  |  |  |  |
| *Pace* |  |  |  |  |
| Walking speed in shorter (10-30s) WBs (m/s) | 0.3 to 0.4 | 0.3 to 0.4 | -0.3 to -0.4 | 0.3 to 0.4 |
| Walking speed in longer (>30s) WBs (m/s) | 0.3 to 0.4 | 0.3 to 0.4 | -0.3 to -0.4 | 0.3 to 0.4 |
| P90 walking speed in WBs >10s (m/s) | 0.3 to 0.4 | 0.3 to 0.4 | -0.3 to -0.4 | 0.3 to 0.4 |
| P90 walking speed in longer (>30s) WBs (m/s) | 0.3 to 0.4 | 0.3 to 0.4 | -0.3 to -0.4 | 0.3 to 0.4 |
| Stride length in shorter (10-30s) WBs (cm) | 0.3 to 0.4 | 0.3 to 0.4 | -0.3 to -0.4 | 0.3 to 0.4 |
| Stride length in longer (>30s) WBs (cm) | 0.3 to 0.4 | 0.3 to 0.4 | -0.3 to -0.4 | 0.3 to 0.4 |
| *Rythm* |  |  |  |  |
| Cadence in all WBs (steps/min) | 0.3 to 0.4 | 0.3 to 0.4 | -0.3 to -0.4 | 0.3 to 0.4 |
| Cadence in longer (>30s) WBs (steps/min) | 0.3 to 0.4 | 0.3 to 0.4 | -0.3 to -0.4 | 0.3 to 0.4 |
| P90 cadence in longer (>30s) WBs (steps/min) | 0.3 to 0.4 | 0.3 to 0.4 | -0.3 to -0.4 | 0.3 to 0.4 |
| Stride duration in all WBs (s) | -0.3 to -0.4 | -0.3 to -0.4 | 0.3 to 0.4 | -0.3 to -0.4 |
| Stride duration in longer (>30s) WBs (s) | -0.3 to -0.4 | -0.3 to -0.4 | 0.3 to 0.4 | -0.3 to -0.4 |
| *Bout-to-bout variability* |  |  |  |  |
| Walking speed bout-to-bout variability in longer (>30s) WBs (%) | 0.3 to 0.4 | 0.3 to 0.4 | -0.3 to -0.4 | 0.3 to 0.4 |
| Stride length bout-to-bout variability in longer (>30s) WBs (%) | 0.3 to 0.4 | 0.3 to 0.4 | -0.3 to -0.4 | 0.3 to 0.4 |
| Cadence bout-to-bout variability (%) | 0.3 to 0.4 | 0.3 to 0.4 | -0.3 to -0.4 | 0.3 to 0.4 |
| Stride duration bout-to-bout variability (%) | 0.3 to 0.4 | 0.3 to 0.4 | -0.3 to -0.4 | 0.3 to 0.4 |

DMO Digital Mobility Outcome; *SPPB* Short Physical Performance Battery; *4m gait speed* Gait speed from the SPPB supervised 4-meter walk test; *Short FES-I* Short Falls Efficacy Scale International; FACIT-F Functional Assessment of Chronic Illness Therapy Fatigue Scale; WBs Walking Bouts.

**Supplementary Table S4.** Comparison of sociodemographic and clinical characteristics between included and excluded participants after PFF surgery.

|  | **Excluded n = 62 (11%)** | **Included n = 505 (89%)** | **p-value** |
| --- | --- | --- | --- |
| **Sociodemographic characteristics** |  |  |  |
| Age, mean (SD) | 77.2 (10.6) | 77.6 (9.4) | 0.717 |
| Gender, female, n (%) | 42 (69%) | 333 (66%) | 0.662 |
| Marital status, married, n (%) | 26 (43%) | 246 (49%) | 0.657 |
| Education less than high school (<10 yrs) / others, n (%) | 34 (55%) | 320 (63%) | 0.663 |
| Recruitment site, n (%) |  |  | <0.001 |
| *Montpellier* | 23 (37%) | 39 (8%) |  |
| *Trondheim / Oslo* | 24 (39%) | 238 (47%) |  |
| *Stuttgart / Heidelberg* | 15 (25%) | 228 (45%) |  |
| Fracture Type, n (%) |  |  | 1 |
| *Femoral neck fracture* | 42 (69%) | 348 (69%) |  |
| *Extracapsular fracture* | 19 (31%) | 157 (31%) |  |
| Days since surgery, median (p25-p75) | 62 (5 - 201) | 60 (23 - 140) | 0.768 |
| Functional Comorbidity Index [0-18], mean (SD) | 3.3 (3.0) | 3.3 (2.1) | 0.411 |
| Walking Aids, n (%) |  |  | 0.002 |
| *Indoor* | 17 (39%) | 253 (62%) | 0.003 |
| *Outdoor* | 28 (64%) | 301 (74%) | 0.144 |
| Recovery Phase, n (%) |  |  | 0.002 |
| *Acute* | 18 (29%) | 100 (20%) |  |
| *Post Acute* | 10 (16%) | 117 (23%) |  |
| *Extended Recovery* | 13 (21%) | 201 (40%) |  |
| *Long Term Recovery* | 21 (34%) | 87 (17%) |  |
| **Clinical characteristics** |  |  |  |
| TUG (s), mean (SD) | 23.7 (17.8) | 19.8 (12.3) | 0.441 |
| SPPB |  |  |  |
| *Total score [0-12], mean (SD)* | 5.6 (3.7) | 6.2 (3.1) | 0.057 |
| *Gait speed (m/s), mean (SD)* | 0.70 (0.50) | 0.70 (0.40) | 0.319 |
| 6MinWT Distance (m), mean (SD) | 278.4 (149.9) | 283.6 (125.9) | 0.777 |
| LLFDI-FC [0-100], median (p25-p75) | 56 (42 - 62) | 49 (42 - 59) | 0.167 |
| Short FES-I [7-28], median (p25-p75) | 8 (7 - 14) | 10 (7 - 14) | 0.257 |
| FACIT-F [0-52], median (p25-p75) | 41 (32 - 47) | 40 (31 - 45) | 0.468 |
| Hearing Impairment [2-8], mean (SD) | 3.2 (1.4) | 2.9 (1.2) | 0.043 |
| Systolic Blood Pressure (mm Hg), median (p25-p75) | 134 (121 - 146) | 132 (120 - 150) | 0.688 |

*Fracture Type:* 1 participant with missing data; *Walking Aids* Acute patients were not included in the analysis as the pre-fracture walking aid usage was assessed. *TUG* Timed Up and Go test; *SPPB* Short Physical Performance Battery; *4m gait speed* Gait speed from the SPPB supervised 4-meter walk test. *6MinWT*  6-Minute Walking Test; *LLFDI-FC* Late Life Function and Disability Instrument – Functional Component; *Short FES-I* Short Falls Efficacy Scale International; *FACIT-F* Functional Assessment of Chronic Illness Therapy Fatigue Scale; *TUG, 5-time chair rise as part of SPPB, 6MinWT and LLFDI* Not performed in acute patients.

**Supplementary Table S5.** Distribution and convergent and divergent validity (correlation coefficients with corresponding 95% CIs) of DMOs in 100 **acute** patients assessed ≤14 days after PFF surgery. Coefficients in bold represent those that matched or exceeded expected values, while coefficients in non-bold represent those that did not match the expected values. Divergent validity coefficients are shown in grey as the sample size was too small.

| **DMO** | **Mean (SD) or median (p25 - p75)** |  | **Convergent** |  | |  | **Divergent** |  |
| --- | --- | --- | --- | --- | --- | --- | --- | --- |
|  |  | **SPPB total** | **4m gait speed** | **Short FES-I** | **FACIT-F** | | **Hearing Impairment** | **Systolic BP** |
| **Walking activity** |  |  |  |  |  | |  |  |
| *Amount* |  |  |  |  |  | |  |  |
| Walking duration (min/day) | 7 (3 - 14) | **0.39 (0.20, 0.57)** | **0.39 (0.22, 0.55)** | -0.08 (-0.27, 0.13) | 0.26 (0.07, 0.44) | | **-0.02 (-0.25, 0.18)** | **0.10 (-0.12, 0.32)** |
| WB step count (steps/day) | 519 (241- 1069) | **0.40 (0.18, 0.57)** | **0.39 (0.20, 0.54)** | -0.08 (-0.29, 0.10) | 0.27 (0.08, 0.43) | | **-0.02 (-0.24, 0.2)** | **0.11 (-0.12, 0.32)** |
| *Pattern* |  |  |  |  |  | |  |  |
| Number of WBs (n) | 57 (59) | **0.41 (0.21, 0.59)** | **0.43 (0.26, 0.58)** | -0.14 (-0.31, 0.07) | 0.28 (0.08, 0.44) | | **-0.06 (-0.26, 0.16)** | **0.11 (-0.13, 0.35)** |
| Number of WBs >10s (n) | 12 (6 - 26) | **0.36 (0.16, 0.54)** | **0.35 (0.16, 0.50)** | -0.06 (-0.26, 0.15) | 0.25 (0.04, 0.41) | | **-0.02 (-0.21, 0.20)** | **0.08 (-0.16, 0.31)** |
| Number of WBs >30s (n) | 1 (0 - 4) | 0.28 (0.04, 0.46) | 0.24 (0.05, 0.43) | 0.06 (-0.15, 0.28) | 0.12 (-0.07, 0.31) | | **0.03 (-0.18, 0.24)** | **0.11 (-0.14, 0.32)** |
| Number of WBs >60s (n) | 0 (0 - 1) | 0.24 (0.03, 0.43) | 0.20 (-0.01, 0.39) | 0.04 (-0.20, 0.27) | 0.09 (-0.11, 0.27) | | **0.01 (-0.19, 0.23)** | **0.11 (-0.17, 0.30)** |
| WB duration (s) | 7.4 (6.6 - 8.3) | 0.04 (-0.15, 0.25) | 0.01 (-0.18, 0.22) | 0.26 (0.05, 0.47) | -0.02 (-0.21, 0.21) | | **0.13 (-0.05, 0.32)** | **0.00 (-0.21, 0.21)** |
| P90 WB duration (s) | 16.8 (12.7 - 22.8) | 0.13 (-0.09, 0.31) | 0.14 (-0.05, 0.32) | 0.17 (-0.06, 0.38) | 0.03 (-0.15, 0.21) | | **0.10 (-0.11, 0.30)** | **0.03 (-0.18, 0.25)** |
| WB duration bout-to-bout variability (%) | 67 (53 - 81) | 0.24 (0.05, 0.42) | 0.22 (0.04, 0.40) | 0.02 (-0.21, 0.22) | 0.11 (-0.07, 0.28) | | **0.00 (-0.19, 0.20)** | **-0.02 (-0.22, 0.21)** |
| **Gait** |  |  |  |  |  | |  |  |
| *Pace* |  |  |  |  |  | |  |  |
| Walking speed in shorter (10-30s) WBs (m/s) | 0.55 (0.08) | 0.07 (-0.16, 0.26) | 0.07 (-0.13, 0.29) | -0.13 (-0.33, 0.06) | 0.10 (-0.11, 0.31) | | **0.06 (-0.14, 0.27)** | **-0.18 (-0.36, 0.05)** |
| Walking speed in longer (>30s) WBs (m/s) | 0.56 (0.09) | **0.31 (0.07, 0.52)** | **0.30 (0.08, 0.49)** | -0.28 (-0.50, -0.05) | 0.19 (-0.04, 0.41) | | **-0.04 (-0.25, 0.18)** | **-0.03 (-0.28, 0.21)** |
| P90 walking speed in WBs >10s (m/s) | 0.62 (0.10) | 0.15 (-0.05, 0.37) | 0.13 (-0.09, 0.32) | -0.13 (-0.31, 0.07) | 0.13 (-0.07, 0.31) | | **0.07 (-0.12, 0.27)** | **-0.17 (-0.38, 0.06)** |
| P90 walking speed in longer (>30s) WBs (m/s) | 0.61 (0.10) | 0.26 (0.03, 0.48) | 0.29 (0.05, 0.48) | -0.24 (-0.44, -0.01) | 0.13 (-0.10, 0.34) | | **-0.03 (-0.25, 0.19)** | **0.02 (-0.22, 0.27)** |
| Stride length in shorter (10-30s) WBs (cm) | 83 (11) | 0.18 (-0.06, 0.37) | 0.17 (-0.04, 0.37) | -0.12 (-0.33, 0.10) | 0.13 (-0.07, 0.31) | | **0.01 (-0.19, 0.20)** | -0.30 (-0.52, -0.06) |
| Stride length in longer (>30s) WBs (cm) | 86 (12) | **0.33 (0.11, 0.52)** | **0.41 (0.21, 0.59)** | -0.29 (-0.50, -0.06) | 0.23 (0.02, 0.46) | | **-0.05 (-0.26, 0.18)** | **-0.08 (-0.30, 0.18)** |
| *Rythm* |  |  |  |  |  | |  |  |
| Cadence in all WBs (steps/min) | 81 (5) | -0.13 (-0.32, 0.10) | -0.08 (-0.28, 0.14) | -0.12 (-0.36, 0.10) | -0.01 (-0.21, 0.18) | | **0.11 (-0.11, 0.30)** | **0.11 (-0.10, 0.33)** |
| Cadence in longer (>30s) WBs (steps/min) | 78 (7) | 0.07 (-0.16, 0.30) | 0.00 (-0.23, 0.24) | -0.14 (-0.37, 0.10) | 0.04 (-0.18, 0.26) | | **0.03 (-0.19, 0.27)** | **0.16 (-0.08, 0.36)** |
| P90 cadence in longer (>30s) WBs (steps/min) | 82 (8) | 0.12 (-0.12, 0.33) | 0.08 (-0.15, 0.31) | -0.09 (-0.33, 0.15) | 0.02 (-0.18, 0.23) | | **0.04 (-0.20, 0.26)** | **0.17 (-0.05, 0.39)** |
| Stride duration in all WBs (s) | 1.37 (0.12) | 0.10 (-0.07, 0.30) | 0.03 (-0.15, 0.23) | 0.12 (-0.08, 0.33) | 0.05 (-0.18, 0.25) | | **-0.04 (-0.24, 0.16)** | **0.02 (-0.20, 0.24)** |
| Stride duration in longer (>30s) WBs (s) | 1.39 (0.20) | -0.01 (-0.22, 0.23) | -0.04 (-0.26, 0.21) | 0.17 (-0.09, 0.40) | 0.05 (-0.19, 0.29) | | **0.07 (-0.14, 0.30)** | **0.04 (-0.18, 0.30)** |
| *Bout-to-bout variability* |  |  |  |  |  | |  |  |
| Walking speed bout-to-bout variability in longer (>30s) WBs (%) | 11 (6) | -0.05 (-0.28, 0.20) | -0.08 (-0.35, 0.18) | -0.01 (-0.26, 0.25) | -0.14 (-0.37, 0.09) | | **0.16 (-0.07, 0.38)** | 0.25 (-0.01, 0.48) |
| Stride length bout-to-bout variability in longer (>30s) WBs (%) | 9 (6) | 0.02 (-0.22, 0.26) | -0.09 (-0.32, 0.16) | 0.05 (-0.18, 0.29) | 0.05 (-0.19, 0.31) | | **0.09 (-0.12, 0.31)** | **0.14 (-0.09, 0.37)** |
| Cadence bout-to-bout variability (%) | 12 (2) | -0.02 (-0.21, 0.19) | 0.04 (-0.16, 0.23) | -0.02 (-0.22, 0.18) | -0.09 (-0.28, 0.13) | | **-0.01 (-0.20, 0.20)** | -0.36 (-0.51, -0.17) |
| Stride duration bout-to-bout variability (%) | 20 (3) | 0.10 (-0.10, 0.29) | -0.09 (-0.28, 0.12) | 0.00 (-0.22, 0.20) | -0.19 (-0.36, 0.01) | | **-0.05 (-0.24, 0.15)** | -0.20 (-0.42, 0.01) |

DMO Digital Mobility Outcome; *SPPB* Short Physical Performance Battery; *4m gait speed* Gait speed from the SPPB supervised 4-meter walk test; *Short FES-I* Short Falls Efficacy Scale International; FACIT-F Functional Assessment of Chronic Illness Therapy Fatigue Scale; *BP* Blood Pressure; WBs Walking Bouts.

Number of acute participants missing DMO values: Walking speed bout-to-bout variability in longer (>30s) WBs and Stride length bout-to-bout variability in longer (>30s) WBs: 27 missing values. Walking speed in longer (>30s) WBs, Maximum walking speed in longer (>30s) WBs, Stride length in longer (>30s) WBs, Cadence in longer (>30s) WBs, Maximum cadence in longer (>30s) WBs, and Stride duration in longer (>30s) WBs: 18 missing values.

**Supplementary Fig. S6.** Known groups validity of all 24 DMOs between the four recovery groups after a PFF. This was partly published previously [1].


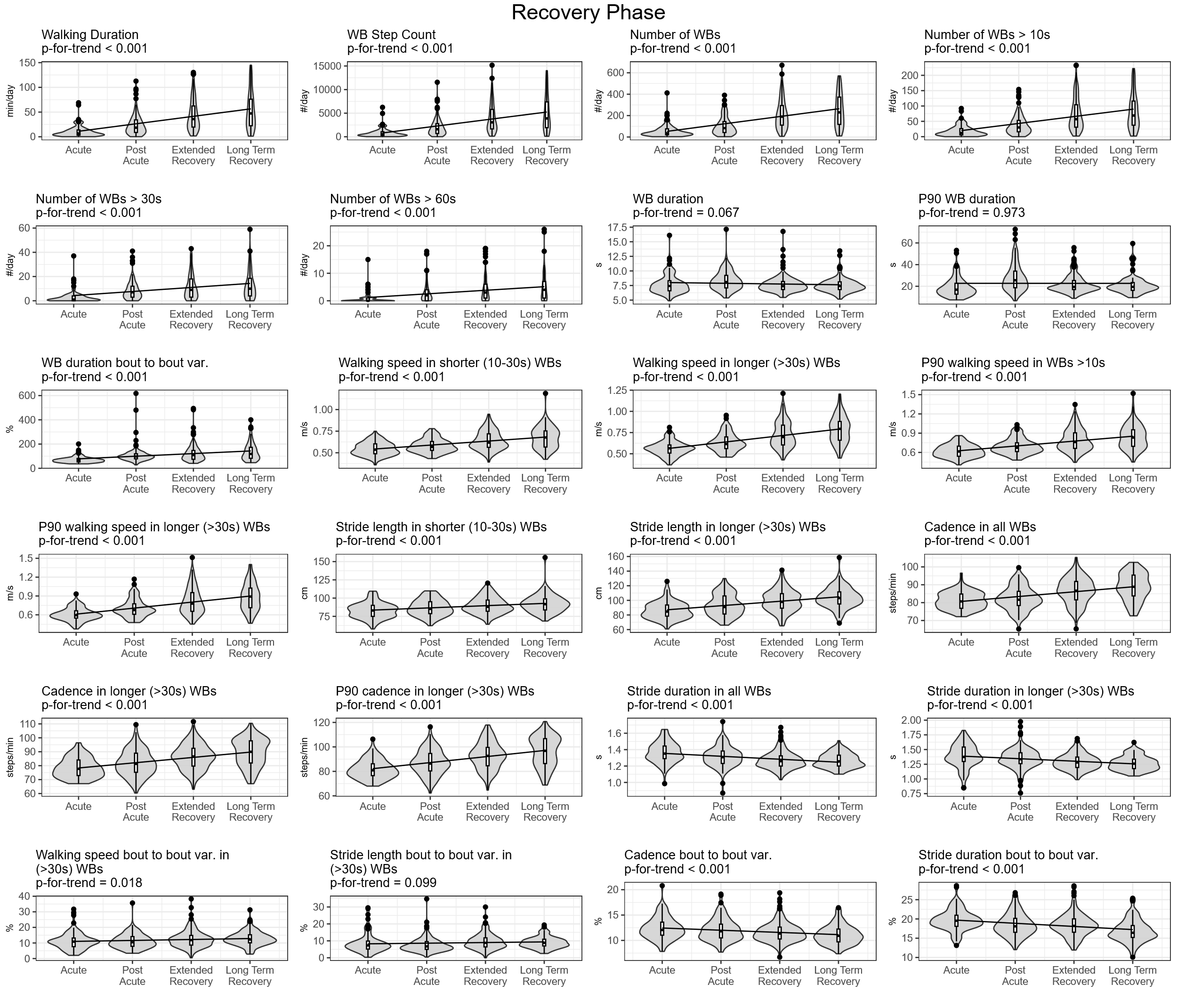


1 Becker, C. *et al.* Digital mobility outcomes to describe real-world walking during recovery from a hip fracture: the Mobilise-D perspective. *European Geriatric Medicine*; 10.1007/s41999-025-01391-w (2026).
